# Incremental cost and cost-effectiveness of low-dose, high-frequency training in basic emergency obstetric and newborn care as compared to status quo: part of a cluster-randomized training intervention evaluation in Ghana

**DOI:** 10.1186/s12992-017-0313-x

**Published:** 2017-12-06

**Authors:** Michelle Willcox, Heather Harrison, Amos Asiedu, Allyson Nelson, Patricia Gomez, Amnesty LeFevre

**Affiliations:** 1 Jhpiego, an affiliate of Johns Hopkins University, 1615 Thames St, Baltimore, MD #200 USA; 2 Jhpiego, 14 Ollenu Rd, Accra, Ghana; 3 Jhpiego, Mamba Point, Monrovia, Liberia; 4 Department of International Health, Johns Hopkins Bloomberg School of Public Health, Johns Hopkins University Global mHealth Initiative, 615 N. Wolfe St, Baltimore, MD USA

**Keywords:** Cost analysis, Economic evaluation, Cost-effectiveness analysis, In-service training, Facility-based training, Low-dose, high-frequency training, Maternal and newborn health, MNH, Frontline health workers, Ghana

## Abstract

**Background:**

Low-dose, high-frequency (LDHF) training is a new approach best practices to improve clinical knowledge, build and retain competency, and transfer skills into practice after training. LDHF training in Ghana is an opportunity to build health workforce capacity in critical areas of maternal and newborn health and translate improved capacity into better health outcomes.

**Methods:**

This study examined the costs of an LDHF training approach for basic emergency obstetric and newborn care and calculates the incremental cost-effectiveness of the LDHF training program for health outcomes of newborn survival, compared to the status quo alternative of no training. The costs of LDHF were compared to costs of traditional workshop-based training per provider trained. Retrospective program cost analysis with activity-based costing was used to measure all resources of the LDHF training program over a 3-year analytic time horizon. Economic costs were estimated from financial records, informant interviews, and regional market prices. Health effects from the program’s impact evaluation were used to model lives saved and disability-adjusted life years (DALYs) averted. Uncertainty analysis included one-way and probabilistic sensitivity analysis to explore incremental cost-effectiveness results when fluctuating key parameters.

**Results:**

For the 40 health facilities included in the evaluation, the total LDHF training cost was $823,134. During the follow-up period after the first LDHF training—1 year at each participating facility—approximately 544 lives were saved. With deterministic calculation, these findings translate to $1497.77 per life saved or $53.07 per DALY averted. Probabilistic sensitivity analysis, with mean incremental cost-effectiveness ratio of $54.79 per DALY averted ($24.42–$107.01), suggests the LDHF training program as compared to no training has 100% probability of being cost-effective above a willingness to pay threshold of $1480, Ghana’s gross national income per capita in 2015.

**Conclusion:**

This study provides insight into the investment of LDHF training and value for money of this approach to training in-service providers on basic emergency obstetric and newborn care. The LDHF training approach should be considered for expansion in Ghana and integrated into existing in-service training programs and health system organizational structures for lower cost and more efficiency at scale.

## Background

In the last 2 decades, under-5 mortality rates have declined by nearly 50% and the rate of decline has increased over the last decade, largely due to increases in coverage and access to essential reproductive, maternal, newborn, and child health (RMNCH) services [1]. Despite these successes in under-5 child health, newborn mortality accounts for 45% of under-5 deaths and reductions in neonatal mortality and stillbirth rates have stagnated with significant gaps in relation to targets, especially in sub-Saharan Africa [1–3]. Globally, an estimated 2.7 million neonatal deaths and 2.6 million stillbirths occurred in 2015, of which 23% of neonatal deaths and 26% of stillbirths occurred during the critical intrapartum period: onset of labor to within 24 h of birth [2, 4–7]. To realize Sustainable Development Goal (SDG) 3—good health and well-being—health systems and implementing partners need to address persistent challenges in providing quality care, but also need evidence on how to most effectively improve care, during the critical pregnancy, intrapartum, and postpartum periods [8].

Ghana has sought to increase access to and affordability of key RMNCH services by expanding national health insurance and providing free maternal care [9]. Despite progress toward the Millennium Development Goals and, more recently, SDG targets, Ghana mirrors some regional positive trends in areas of stunting and wasting but still faces significant challenges in improving newborn health outcomes which is reflected in low levels of progress in skilled birth attendance, neonatal mortality, and under-5 mortality [10]. Among under-5 deaths in Ghana, 40% occurred during the neonatal period in 2012 [1]. For every 1000 live births in Ghana, 28 children die within the first 28 days of life, more than twice the SDG target of 12 per 1000 live births [1]. Despite global declines in absolute numbers of stillbirths of 19% from 2000 to 2015, the absolute number of stillbirths within Ghana only reduced 1.8% over the same time period; the country stillbirth rate was 22 per 1000 total births in 2009 [2, 11]. Recognizing these gaps in progress, the Ghana Health Service (GHS) and Ministry of Health are actively pursuing strategies to improve quality of RMNCH care with focus on a better-trained workforce and improved rural access to quality labor, delivery, and newborn health services [12].

### Low-dose, high-frequency (LDHF) training

Clinical care quality is strengthened by increased knowledge, acquired skills, and peer consultation; training in many forms has long been utilized to improve clinical performance and, consequently, RMNCH outcomes [13]. The best and most cost-effective methods of training in low-resource settings, however, continue to be tested. Traditionally, training in low-resource settings has assumed a workshop-based approach wherein a subsample of providers are removed from their clinical environment and trained off site for an intensive period of time. Workshop training may improve attendees’ capacity, but knowledge and skills may not be transferred to other co-workers at the facility nor translated into provider practice or performance [14, 15]. Various methods for continuing medical education and in-service training have established strong evidence on best practices for learning: repetition is correlated to higher retention of knowledge and skill, having the same setting for training as clinical practice is correlated with higher gains in skills and performance, simulation-based practice and interactive methods are more effective than didactic lecture [16–19].

Low-dose, high-frequency (LDHF) is an approach to improve the in-service training model to better enhance the health workforce capacity, sustain health provider gains in knowledge and skills, and help learners translate clinical lessons into practice [20]. The concepts and terminology of on-site short teaching lessons (low-dose) followed by longer practice sessions (high-frequency) have been applied in different ways and settings from maternal and newborn health trainings by Jhpiego in Uganda to pediatric cardiopulmonary resuscitation training in the Children’s Hospital of Philadelphia in the United States [14–16, 21]. LDHF educates with small quantities of content, frequent repetition, increased interaction, simulation-based practice, and supervised direct patient care that keeps providers in the facility environment while learning. Jhpiego has combined evidence with extensive training experience to create an LDHF approach that better targets and supports the health workforce with competency-based education rather than solely focusing on knowledge improvement [22]. The LDHF training program in Ghana was designed after a facility quality improvement project for child health, Project Fives Alive!, identified that in aiming to reduce under-5 mortality, an additional focus on improving clinical skills for quality delivery and postpartum services was necessary to reduce the large proportion of under-5 deaths that occur on the day of birth [23]. The LDHF training implemented in Ghana aims to accelerate newborn survival by improving health provider capacity to provide quality institutional delivery and postpartum services at the facility.

### Study objectives

This study examines programmatic costs and health effects, calculated by DALYs averted, of LDHF training on institutional stillbirth rates and on newborn survival outcomes of 40 facilities included in the impact evaluation in three regions of Ghana from 2014 to 2017 and compares the incremental cost-effectiveness of LDHF training with the alternative of no training (the status quo).

## Methods

### Study setting

Of the 24.7 million people that live in Ghana, 21.4% are in the Central, Western, and Upper West regions where the LDHF training program was jointly planned with the Ghana Health Service and Ministry of Health and coordinated so as not to overlap with existing programs [24]. Utilization of key maternal health care services across Ghana has increased, but delivery at a health facility has not seen the same levels of utilization as other services [25]. In 2011, 87% of pregnant women had four or more antenatal care visits and 83% had a postnatal care visit within 2 days of birth, but only 68% of live births had a skilled attendant at delivery, and key health service utilization is lower in rural populations than in urban [1, 9]. The Central and Western regions have larger proportions of their population in urban areas, 47.1% and 42.4%, respectively, than Upper West region, where 16.3% of population is urban [24]. In the study regions, public and mission hospitals were selected as LDHF training sites if they averaged 30 or more births per month and had at least three skilled birth attendants on staff who actively provided labor, birth, and immediate postnatal care at the facility.

### Program description

The LDHF training program was adapted from a 2-week classroom-based basic emergency obstetric and newborn care (BEmONC) package to two 4-day low-dose onsite trainings. The LDHF trainings incorporated high-quality onsite instruction with a whole-team perspective, clinical simulation-based practice with models, mentorship and coaching, and a daily lunch. Following the LDHF trainings, content is reinforced through high-frequency mentorship: individual phone calls between regional GHS master mentors (MMs) and trainees, peer practice with medical models in the facility, and text message reminders and quizzes. Nongovernmental organization (NGO) staff contacted the trained MMs, peer practice coordinators, and providers to ensure mentoring and practice were continuing after the LDHF onsite training and also monitored the mobile messaging system to make sure text messages sending appropriately. In contrast to traditional training models, which train a small subpopulation of health workers outside of the clinical setting, in the LDHF model all midwives and nurses providing labor, delivery, and immediate postpartum care at a participating facility have the opportunity to receive the same training.

Program activities are understood through three phases: Development—adaptation of the curriculum from workshop to LDHF training approach; Startup—planning and conducting MM trainings, health information officer trainings, and regional health team meetings; and, finally, Implementation—organizing and conducting LDHF trainings in two main sessions (LDHF1 to cover basic care and LDHF2 for complications) with provider knowledge and skills assessments, and initiating mobile mentoring (mMentoring) and peer practice. Additional details on the program and health effects measurement are found in the Accelerating Newborn Survival project impact evaluation [26]. A full program description with timeframes for each phase is found in Table 1.

**Table 1:** Program description

Program Activity Category by Phase	Description	Inputs	Time
Development			4 months
Curriculum adaptation	Format BEmONC materials into Learning Resource Package for LDHF format	Copy editing fees, technical assistance for adapting curricula	4 months
Start Up			8 months
Health Information Officer Training	3-day course for HIOs of participating health facilities to train on data collection and validation of maternal and newborn health indicators	Transportation, Accommodation, materials, food, facilitator costs	3 days per training
Master Mentor: Training of Trainers	8-day course to review and confirm expertise in content; held one centralized training in each region for which 10 MMs selected by Regional Health Team	Transportation, Accommodation, materials, food, facilitator costs	8 days per training
Regional Health Team Meetings	1-day lunch meeting to share program objectives and target facilities of the region for participation	Transportation, lunch, materials	1 month for all 3 regions
Training Equipment	Procurement of patient models, data collection tools, medical equipment, printed manuals and training materials	Items needed per training team	3 months
Implementation			6 months per Wave
LDHF 1 facility based training	First LDHF workshop: basic delivery and newborn care; Peer Practice Coordinator training	Transportation, Accommodation, materials, food, facilitator costs	1 week
LDHF 2 facility based training	Second LDHF workshop: emergency and delivery complications for maternal and newborn care	Transportation, Accommodation, materials, food, facilitator costs	1 week, 1 month after LHDF1
mMentoring Phonecalls	Hierarchy of phone calls to discuss skills practice, cases, and any questions that arise by trainees	Phone calls made to recipients, duration of call	6 months
mMentoring Text Messages	Automated practice reminders and interactive SMS based quiz questions on trained content	Telerivet system license, phone for sending messages, phone calls	6 months
Master Mentor: Refresher Training	8-day workshop refresher course on content, education, and mentorship skills	Transportation, Accommodation, materials, food, facilitator costs	8 days per training
Recurrent			
Personnel	Staff time during each phase separate from activity specific valued inputs for travel and trainings to separate indirect personnel cost from activity cost		Recurrent
Buildings & Maintenance	Local office and overhead costs such as utilities		Recurrent
Furniture & Equipment	Office furniture and equipment used for program activities by staff		Recurrent

### Cost data

Assuming a 3-year analytic time horizon corresponding to the program’s length, data on economic costs were collected for all program activities based on a review of program financial records, including purchase orders, original receipts kept on record in the finance department, and recurring overhead allocation reports. Cost details were confirmed and supported by interviews with the LDHF implementation team, especially important because many program costs were summarized and reported at the regional rather than the activity level. Personnel salaries were increased per fiscal year based on informant interviews and human resources records, and applied monthly to appropriate phases during which staff were supporting the program. Market prices or regional price averages were used to value the furniture, equipment, and other resources used by LDHF program staff. Operating costs were included for each phase, by time period and value of personnel effort, building space, maintenance and utilities, and furniture and equipment used by the LDHF training program.

*Development costs* included costs associated with adapting the BEmONC curriculum, including personnel time and editing services to develop the learning resource package of materials: facilitator and learner manuals, slides for instruction, and standardized assessments. *Startup costs* included all inputs related to regional health team meetings and trainings for health information officers and MMs. The main inputs were transportation, program staff per diem and resource personnel fees, and food and lodging costs associated with the necessary travel*. Implementation costs* included all inputs related to the LDHF trainings and mMentoring follow-up. These similarly included transportation, personnel, and accommodations costs but also trainee-associated costs of lunch and training materials as well as equipment and expendables costs for simulation-based trainings, including practice models of MamaNatalie™ and NeoNatalie™, medical equipment, examination gloves, face masks, and cleaning supplies.

Key cost model assumptions are as follows: 1) once averaged from multiple data sources or direct from one single and validated source, a cost is stable and representative of further repeated activities at the facility or regional level, 2) key development and startup costs such as curriculum development were allocated across all facilities that received LDHF training, 3) personnel time and salary cost were measured monthly and then averaged based on the corresponding timeline of each LDHF program phase so as to have one personnel cost for each staff member during each phase, 4) expendable training items are costed based on either facilities or region depending on rate of use and reusability as informed by the LDHF clinical training team. The program costs collected represent Jhpiego’s LDHF training program reach to three regions, 40 facilities, and 428 health workers receiving training.

### Health effects data

The impact evaluation of the LDHF training intervention on newborn survival measured health effects by tracking institutional newborn mortality within 24 h of birth or until discharge and institutional intrapartum stillbirths; indicators were tracked at the facility with supplemental registers in 1-month increments and pooled as 6-month average rates [26]. Each tracking period—the 6 month baseline prior to LDHF training, 1–6 months after LDHF training, and 7–12 months after LDHF training—included over 30,000 live births from the study sites in the three regions (38,192; 36,160; and 31,498 respectively). Early newborn mortality reduced 48% from baseline during the 1–6 months immediately following the LDHF training, and reduced 56% from baseline in the 7–12 months period [26]. Intrapartum stillbirths reduced 35% from baseline during the 1–6 months immediately following the LDHF training, and reduced 49% from baseline during the 7–12 months period [26]. Data were recorded by the service providers and verified by study staff. Service providers and facility-based health information officers were trained on the use of supplemental registers, new indicators, and data quality. Aside from these trainings, the study team had minimal onsite presence and clinical practice was continued as usual.

Disability-adjusted life years (DALYs) were calculated in this analysis using estimated lives saved, average life expectancy in Ghana, no age weighting, omitting years lived with disability (YLD), and discounting at 3% so as to discount health effects at the same rate as costs [27]. Unlike the impact of chronic illness or cancer which is heavily influenced by years lived with disability (YLD), the majority health effect impact from newborn mortality is derived from years of life lost [28, 29]. The Global Burden of Disease 2016 valuation of DALYs also reflects this difference: neonatal disorders are the leading cause of death under-5 years but only the fourth contributing cause of YLD, the ratio of all age YLD to DALYs remains low throughout sub-Saharan Africa, and only a quarter of the DALY counts for Ghana are estimated to be from YLD [30, 31]. The omission of YLD for this analysis will make the results slightly more conservative as we may be underestimating the full health impact of improving newborn care as related to morbidity, but it will not considerably change the overall findings of cost-effectiveness.

The cost and health effect data were collected from existing records from the implementation of the LDHF training and from the impact evaluation; the cost-effectiveness analysis using this data was determined nonhuman subjects research by the Johns Hopkins Bloomberg School of Public Health Institutional Review Board.

### Analysis

The analysis of cost data was conducted in Excel. All costs are presented in 2015 US dollars. All capital costs are discounted at 3% and one-time development and startup activities annualized for the analytic time horizon of 3 years, matching the actual program timeframe [27]. The activities and costs included in this analysis are from the programmatic perspective. Although we assume that personnel and overhead costs would be much lower if the program were adapted, continued, or supported by a local partner or government, we kept the wide range of actual Jhpiego cost so as to get the most accurate result for incremental cost-effectiveness. We did not include societal costs to beneficiaries as the cost of care seeking is separate from the cost of training health care workers to provide quality care; in this manner, costs to beneficiaries would remain the same whether or not LDHF training was provided. Furthermore, the programmatic perspective can be useful to decision-makers that are weighing benefits with costs and striving to build balanced health systems in the midst of ever increasing health expenditure as a share of the economy [32].

To determine the incremental cost-effectiveness of program activities, a simple decision tree was created using TreeAge Pro 2017 software comparing incremental cost and effects of LDHF training to no training [33]. Base case results of costs and health effects were explored with both univariate sensitivity analysis of each cost category and probabilistic sensitivity analysis with sampling from the expected ranges of costs and effects using the Monte Carlo simulation. All programmatic costs were considered for all waves of implementation. High and low values for each activity were established by taking the maximum option for each input and the minimum option for each input. When multiple data points were not available for a particular cost input, the base case value was inflated or deflated by 25%. The health effects high and low were determined from the best case and worst case scenario based on the standard deviation from the base health effects findings of reduced early newborn mortality and intrapartum stillbirth rates. To conduct a probabilistic sensitivity analysis, distributions were applied to base case parameters which significantly affected the results in univariate analysis. Uncertainty in the results was explored through tornado diagrams, cost-effectiveness planes, and cost-effectiveness acceptability curves [27, 34, 35].

### Essential LDHF program model and comparison to workshop training

As part of sensitivity analysis, we tested model variations and developed a major set of modifications through discussion with the LDHF program team to explore sustainability and changes in the LDHF program activities if continued or implemented within the Ghana health system. Assumptions were tested together to assess the cost of the essential activities of the LDHF training program implementation in a sustainable scenario that maximizes efficiencies. This Essential LDHF Program scenario was then compared to a traditional BEmONC workshop training hosted by the Ghana College of Nurses and Midwives (GCNM). The GCNM conducted three workshops on BEmONC targeting educational institution tutors and their teaching facility lead clinical preceptors. Two-week-long workshops were conducted in Accra and Kumasi: one week of classroom-based learning and a second week of field site coaching and hands-on experience, both facilitated by GCNM staff and guest trainers. Follow-up was conducted mainly via phone calls to all trained providers; however, one onsite follow-up visit was conducted to create a case study of how the training had influenced the participating providers. Costs were collected for the traditional workshop with the same methods used to collect the LDHF training costs.

## Results

The full cost of the LDHF training program for all 40 facilities over the 3-year analytic time horizon in three regions was $823,134. The mean total cost per facility was $20,578 including $114 for development (0.6% of total), $695 for startup (3.4% of total), and $19,769 for implementation (96.1% of total). As an average of 11 health care providers participated at each facility, the total program mean cost per provider trained is $1974.

Personnel (44% of overall cost) and the LDHF trainings held as two separate facility-based visits were the major drivers of the program costs: 20% of overall cost for LDHF1 and 14% for LDHF2 (Table 2). The second LDHF facility-based training was less costly due to shorter duration and use of previously provided medical models for peer practice.

**Table 2:** Program costs by wave and total program

Cost Category	Wave 1 (USD)	Wave 2 (USD)	Wave 3 (USD)	Wave 4 (USD)	Total Program Cost	% of Total Cost
Development						
Investment Costs						
Curriculum adaptation	$224	$358	$179	$134	$896	0.11%
Furniture & Equipment	$275	$439	$220	$165	$1099	0.13%
Recurrent Costs						
Personnel Salaries	$384	$615	$301	$226	$1527	0.19%
Buildings, Maintenance, Overhead	$261	$417	$209	$157	$1044	0.13%
*Total Development Costs*	*$1144*	*$1830*	*$909*	*$682*	*$4564*	*0.55%*
Start Up						
Investment Costs						
Curriculum adaptation	$448	$716	$358	$269	$1791	0.22%
Regional Health Team Meetings	$29	$46	$23	$17	$115	0.01%
Master Mentor: Training of Trainers	$952	$1523	$762	$571	$3808	0.46%
Health Information Officer Training	$168	$268	$134	$101	$671	0.08%
Furniture & Equipment	$549	$879	$439	$330	$2197	0.27%
Training Equipment	$788	$1261	$631	$473	$3153	0.38%
Recurrent Costs						
Personnel Salaries	$2791	$4465	$2186	$1639	$11,080	1.35%
Buildings, Maintenance, Overhead	$1250	$2000	$1000	$750	$5000	0.61%
*Total Start Up Costs*	*$6975*	*$11,159*	*$5533*	*$4150*	*$27,816*	*3.38%*
Implementation						
Investment Costs						
Curriculum adaptation	$3895	$3895	$3895	$3895	$15,582	1.89%
Regional Health Team Meetings	$251	$251	$251	$251	$1002	0.12%
Master Mentor: Training of Trainers	$8282	$8282	$8282	$8282	$33,128	4.02%
Health Information Officer Training	$1459	$1459	$1459	$1459	$5836	0.71%
Furniture & Equipment	$4779	$4779	$4779	$4779	$19,116	2.32%
Training Equipment	$2365	$2365	$2365	$2365	$9460	1.15%
Recurrent Costs						
Personnel Salaries	$68,676	$102,855	$97,454	$92,052	$361,037	43.86%
LDHF 1 facility based training	$42,188	$66,938	$34,241	$23,656	$167,024	20.29%
LDHF 2 facility based training	$30,981	$47,212	$23,585	$16,700	$118,478	14.39%
mMentoring Text Messages	$522	$512	$513	$513	$2061	0.25%
mMentoring Phonecalls	$374	$366	$366	$366	$1473	0.18%
Master Mentor refresher training	$5564	$7822	$4812	$2257	$20,456	2.49%
Buildings, Maintenance, Overhead	$8818	$8818	$8818	$8818	$35,272	4.29%
*Total Implementation Costs*	*$178,983*	*$255,555*	*$190,820*	*$165,395*	*$790,753*	*96.07%*
TOTAL COSTS	$187,101	$268,545	$197,261	$170,226	$823,134	

### Cost-effectiveness

With deterministic calculation under which all inputs remain constant and non-random, the base case cost-effectiveness of the LDHF training program is $1497.77 per life saved or $53.07 per DALY averted.

### Sensitivity analyses

**Fig. 1:**
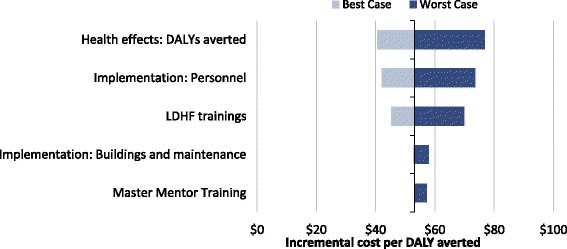
Tornado diagram: One way sensitivity analysis top five most impactful model components on the overall ICER

With deterministic calculations for high and low case sensitivity analyses, we established a best case with measures of high effectiveness and lower limit costs. The best case showed a total program cost of $387,808 and a total of 20,313 DALYs averted; this translates to an incremental cost of $19.09 per DALY averted. In the converse, worst-case-scenario measures of low effectiveness and upper limit costs, the total program cost $1,596,116 with a total of 10,707 DALYs averted; this translates to an incremental cost of $149.08 per DALY averted (Table 3). One-way analysis of key parameters shows that the LDHF training program’s effectiveness, personnel cost, and resources needed for facility-based trainings are the parameters which most impact the incremental cost per DALY averted (Fig. 1).

**Table 3:** Incremental cost effectiveness of LDHF training in 3 regions, with 40 facilities v No Training

	Base Scenario	Best Case (high effectiveness, lower limit cost)	Worst case (low effectiveness, upper limit cost)	Probabilistic (95% CI)	Essential LDHF Model
Total LDHF Program Cost	$823,134	$387,808	$1,596,116	$831,575 ($389,041 – $1,542,848)	$224,026
Total DALYs averted	15,510	20,313	10,707	15,562 (10778–20,192)	10,707
Cost per DALY averted	$53.07	$19.09	$149.08	$54.79 ($24.42 - $107.01)	$20.92

**Fig. 2:**
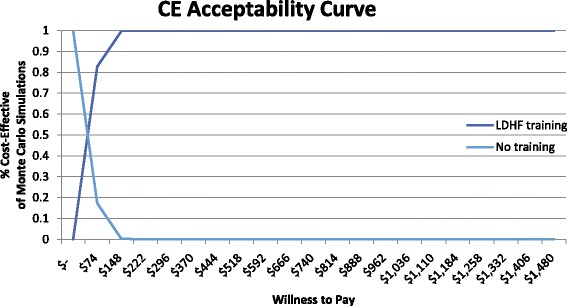
Incremental cost-effectiveness acceptability curve of LDHF training v No training

**Fig. 3:**
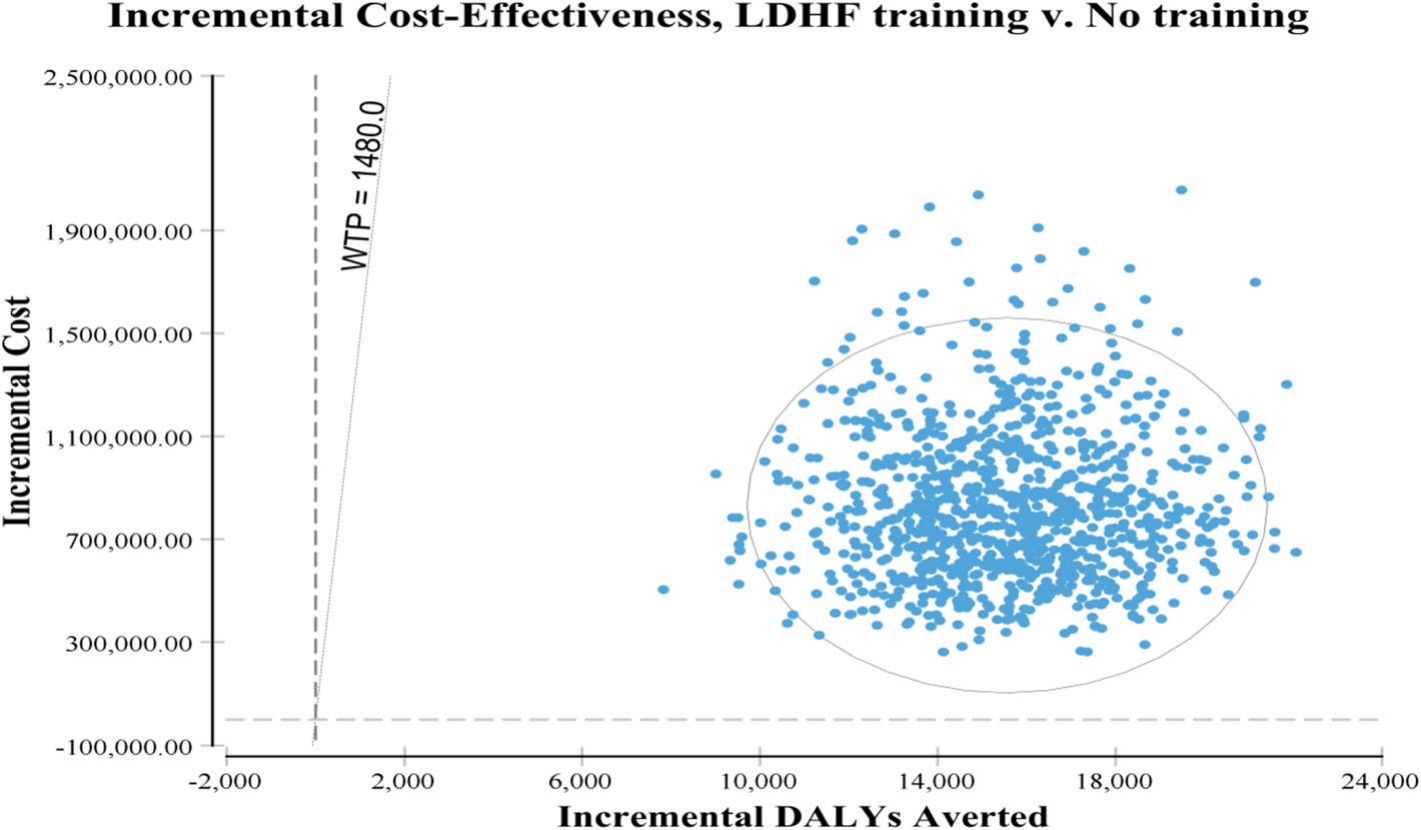
Cost-effectiveness plane of LDHF Training v Status quo in three regions of Ghana valued at the willingness to pay of $1480 GNI per capita in Ghana

Probabilistic sensitivity analysis was conducted to sample from within key cost distributions and effects distributions to better evaluate the robustness of the base case findings (Table 4). Under the probabilistic sensitivity analysis, the mean incremental cost-effectiveness ratio of $54.79 per DALY averted (median $50.68, 95% CI $24.42–$107.01) suggests the LDHF training program as compared to no training has 100% probability of being cost-effective above a willingness to pay threshold of $1480, which was Ghana’s gross national income (GNI) per capita in 2015 (Fig. 2). Each output from the Monte Carlo simulation can be seen plotted in the cost-effectiveness quadrant of increased health effect and increased cost and all simulation results fall under the willingness to pay threshold of $1480 (Fig. 3). The GNI or gross domestic product (GDP) per capita— $1442 in Ghana in 2015 — is a standard measure which helps establish a standard threshold by which to assess interventions, though it is not indicative of affordability or funding [36–38]. Under the GDP or GNI per capita thresholds, 100% of the probabilistic sensitivity analysis estimates of incremental cost per DALY averted by the LDHF program would be considered highly cost-effective in Ghana.

**Table 4:** Sensitivity Analysis parameters for one way, multivariate, and probabilistic sensitivity analysis

Parameters	Deterministic Base case (Low – High)	Probabilistic mean value (95% CI)	Distribution Type	α	λ
Development					
*Investment*					
Curriculum adaptation	$896 ($640 – $1221)	n/a, remain as in base case			
*Recurrent*					
Personnel Salaries	$1527 ($848 - $2529)	n/a, remain as in base case			
Buildings, Maintenance, Overhead	$1044 ($421 - $1769)	n/a, remain as in base case			
Furniture & Equipment	$1099 ($632 -$2378)	n/a, remain as in base case			
Start Up					
*Investment*					
Curriculum adaptation	$1791 ($1279 - $2442)	n/a, remain as in base case			
Health Information Officer Training	$671 ($399 - $1389)	n/a, remain as in base case			
Master Mentor: Training of Trainers	$3808 ($1854 - $7825)	n/a, remain as in base case			
Regional Health Team Meetings	$115 ($55 - $217)	n/a, remain as in base case			
Training Equipment	$3153 ($693 - $14,077)	$3024 ($0 - $22,856)	Gamma	0.20	6.21 E-05
*Recurrent*					
Personnel Salaries	$11,080 ($6042 - $18,561)	$10,930 ($2096 - $24,946)	Gamma	3.09	2.79 E-04
Buildings, Maintenance, Overhead	$5000 ($597 - $13,589)	$5032 ($19 - $22,222)	Gamma	0.57	1.15 E-04
Furniture & Equipment	$2197 ($1264 - $4756)	n/a, remain as in base case			
Implementation					
*Investment*					
Curriculum adaptation	$15,582 ($9569 - $23,120)	$15,658 ($4979 - $32,421)	Gamma	5.27	3.38 E-04
Health Information Officer Training	$5836 ($2985 - $13,151)				
Master Mentor: Training of Trainers	$33,128 ($13,869 - $74,084)	$33,682 ($1875 - $120,093)	Gamma	1.16	3.50 E-05
Regional Health Team Meetings	$1002 ($413 - $2054)	n/a, remain as in base case			
Training Equipment	$9460 ($2309 - $39,416)	$9,6129 ($0 – $65,749)	Gamma	0.23	2.44 E-05
LDHF 1 facility based training	$167,024 ($85,256 - $309,243)	$173,400 ($21,445 - $745,323)	Gamma	2.18	1.30 E-05
LDHF 2 facility based training	$118,478 ($57,877 - $217,833)	$116,992 ($18,514 - $305,365)	Gamma	2.17	1.82 E-05
mMentoring Phonecalls	$1473 ($631 - $2941)	n/a, remain as in base case			
mMentoring Text Messages	$2061 ($1141 - $3302)	n/a, remain as in base case			
Master Mentor: Refresher Training	$20,456 ($10,381 - $46,288)	n/a, remain as in base case			
*Recurrent*					
Personnel Salaries	$361,037 ($169,236 - $658,141)	$364,675 ($46,819 - $1,005,255)	Gamma	2.15	5.95 E-06
Buildings, Maintenance, Overhead	$35,272 ($9417 - $89,609)	$35,060 ($360 – $159,554)	Gamma	0.74	2.11 E-05
Furniture & Equipment	$19,116 ($9453 - $45,024)	$19,534 ($596 – $66,203)	Gamma	1.08	5.65 E-05
Health Effects	Deterministic Base case (Low – High)	Probabilistic (95% CI)	Distribution Type	Mean	SD
Reduction in Newborn Mortality rate (per 1000 live births) from baseline compared to 1–6 months after LDHF training	48% (37% - 60%)	48.0% (25.5% - 71.7%)	Normal	48.1%	11.5%
Reduction in Newborn Mortality rate (per 1000 live births) from baseline compared to 7–12 months after LDHF training	56% (39% - 73%)	56.0% (22.4% - 92.1%)	Normal	56.0%	17.1%
Reduction in Stillbirth Rate (per 1000 births) from baseline compared to 1–6 months after LDHF training	35% (22% - 48%)	35.2% (10.8% - 58.8%)	Normal	34.8%	12.9%
Reduction in Stillbirth Rate (per 1000 births) from baseline compared to 7–12 months after LDHF training	49% (33% - 65%)	49.1% (18.2% - 79.4%)	Normal	49.0%	15.8%

### Essential LDHF program model comparison to workshop training costs

The traditional workshop approach costs $901 per provider compared to $506 per provider trained on average under the Essential LDHF Program Model, which accounts for expected changes in costs and efficiencies for the program implementation (Table 5).

**Table 5:** Modeled Essential LDHF Program cost per trainee (nurses and midwives) of LDHF rollout in 40 sites over 4 Waves and BEmONC classroom based workshops as implemented by Ghana College of Nurses and Midwives (GCNM)

Cost Category LDHF	Cost per trainee (USD)	Cost Category Workshop GCNM	Cost per Trainee (USD)
Number of Providers Trained	428		64
Development LDHF Training	$2	Development BEmONC Workshop	$75
Start Up	$8	Start Up	$101
Implementation	$497	Implementation	$724
Total program	$506	TOTAL PROGRAM	$901

Assumptions for Modeled Essential LDHF Activities Cost

1. Master mentor training conducted by external consultants Master mentoring training would be conducted solely by resource personnel paid standard honorariums rather than by NGO personnel.

2. Resource personnel / consultants would continue to lead and assist in trainings to help Master Mentors assume role of lead trainers; however, their involvement would taper and MMs would continue to lead trainings independently.

3. Health information officer trainings to discuss data collection and health registers were not included in the government led scenario developed under the assumption that this was a research task with focus on providing accurate data back to NGO. Similarly, Regional health officials’ meetings to discuss the LDHF program were not included.

4. Duration of onsite LDHF trainings were reduced to three days under the assumption that full OSCE assessments may not be conducted in programs at scale and for government led continuing medical education initiatives.

5. Peer practice coordinators would not receive an additional day of training and would integrate explanation of the role during the existing days of LDHF training.

6. Personnel costs would decrease as temporary consultants are contracted as clinical trainers and are not salaried program staff. Clinical team time spent on program management was reduced except for 10% of technical expert time, one full time program officer, and a portion of admin and finance time devoted to the LDHF program.

## Discussion

### LDHF approach components

The initial investments needed to begin a new approach to training—such as adaptation of the standardized BEmONC training content into an LDHF learning package, the MM training, and procurement of teaching equipment—all contribute to each facility and health provider that receives that LDHF training. Though the costs of these activities alone are significant, development and startup costs were, together, less than 4% of total program cost because they are shared across all facilities that use the LDHF approach with MMs to lead the training. The main cost drivers are the resources expended to support program personnel and the inputs for onsite LDHF trainings during the implementation phase. Any changes to personnel structure or salaries have a significant impact on the program’s overall cost. LDHF trainings, both the first training that focuses on basic delivery and the second training that focuses on complications and emergency care, have many constants in terms of cost but are sensitive to changes in number of days and number of support staff.

A common expectation is that moving from hotel-based traditional 1- or 2-week workshops to onsite trainings will be less expensive; however, onsite training is not guaranteed to be less expensive though there are key differences in cost drivers. Cost drivers of LDHF onsite trainings are travel and accommodation of MMs, the clinical training team, and support staff; in contrast, much of the cost for traditional workshops is for travel and accommodation for the training recipients. In Ghana, the LDHF onsite training setting enables capacity-building for the full workforce responsible for maternal and newborn health (MNH). This enhances the base capacity of many providers and the facility as a whole, rather than sole reliance on a single trained colleague who may or may not be present when needed.

The LDHF program and lessons learned directly support Strategy 6, “Improving the capacity of facility-level health workers to address newborn care,” as set forth in the National Newborn Health Strategy and Action Plan 2014–2018 by the Ministry of Health, Ghana [12]. Through this LDHF approach, Jhpiego has identified one approach to optimal delivery of “in-service competency-based training” on essential obstetric and newborn care for midwives in hospitals and Community-Based Health Planning and Services facilities [12]. Project Fives Alive! demonstrated successful scale-up of systems strengthening efforts, engaging over half of Ghana’s districts through key learning and quality improvement collaborations [23]. Future training programs could follow a similar path to scale and strengthen the LDHF approach to training and leverage existing stakeholder partnerships.

### Cost-effectiveness of LDHF training as compared to no training

How do we judge the value for money of this new approach? The impact evaluation of the LDHF training showed that this approach led to increased competency, retained knowledge and competency at 1 year, and associated health effects of decreased newborn deaths and stillbirths at the facility [26]. These effects show that this LDHF approach, with onsite training, peer practice, a low-cost mobile messaging system, and mentorship follow-up by phone, was good value for the money expended as measured by the population health impact in Ghana. The LDHF training program saved an estimated 262 lives based on the reductions seen in newborn deaths at the facility in the first 24 h or before discharge; in addition, an estimated 288 lives were saved based on reductions seen in the rate of institutional intrapartum stillbirths. At 28.2 DALYs per death averted, the program in total averted 15,510 DALYs in the base case scenario, at a cost of $53.07 per DALY averted. By using actual NGO cost, matching the analytic time horizon to the project timeframe of 3 years, and not assuming continued health effects for years beyond the project’s end, the base case cost per DALY is conservative. As the impact evaluation established retention of provider competency and continued improvements at 1 year following LDHF training, improvements in lives saved could be assumed beyond the year of data collected, resulting in greater cost-effectiveness in decreased cost per DALY averted.

We tested the model with univariate, multivariate, and probabilistic sensitivity analyses to better understand how our results would change in relation to changes in key parameters. The cost-effectiveness results were mainly affected by changes in effectiveness of the LDHF training program in improving newborn health outcomes, changes to personnel structures or salaries, or changes to LDHF training costs. In the multivariate deterministic calculation of best case and worst case, the cost per DALY averted ranged from $19.09 to $149.08; in the probabilistic sensitivity analysis, the mean cost per DALY was very similar to the base case but the range was slightly smaller, $24.42–$107.01. All scenarios simulated to test incremental cost-effectiveness of LDHF training compared to no training were highly cost-effective when assessed with the threshold of GNI per capita, $1480 in Ghana in 2015 [39].

In a *Lancet* priority-setting article of 2006, low-cost opportunities for sub-Saharan Africa in MNH care interventions—such as increasing primary care coverage, care quality, and targeted neonatal packages—range from $82 to $409 per DALY averted [40]. A 2016 comprehensive look at returns on investment evaluated the cost per DALY ranges for several interventions designed to decrease maternal and newborn morbidity and mortality—participatory women’s groups, training of midwives or village health workers, and safe motherhood and facility interventions—and found that they ranged from $150 to $1000 per DALY averted [41]. The LDHF training cost per DALY averted at base case falls lower than either of these ranges, indicating it may be preferable to other MNH interventions in achieving population health impact at less cost. However, though per capita measures such as GDP or GNI are widely used to standardize cost-effectiveness evidence, using these measures alone to assess value for money can inhibit decision-makers in understanding trade-offs and relating cost to their local health budget or resource-restricted context [36, 42]. Ghana has health financing challenges relevant to the possible continuation of LDHF training. Funds approved for MNH do not always reach the health sector and are frequently delayed even when released; total government spending on health per capita, $35 in 2014, still falls below the estimated amount necessary to achieve basic health service package delivery [43, 44]. As public spending in Ghana is only 53% of total health expenditure and the total health expenditure per capita, $146 in 2015, is modeled to increase only to $218 by 2030, scaling a new training program as it was implemented by an NGO may be cost prohibitive [45, 46]. We created a cost model for Essential LDHF Program activities that would estimate the cost-effectiveness of an LDHF program that was integrated into existing health system management structures. LDHF program experts defined the major assumptions for this scenario and found that, on average, it would cost $506 per provider for LDHF training (Table 5). Using the modeled program cost of $224,026 for the 40 facilities in three regions and the lower limit of health effects, the incremental cost would be $20.92 per DALY averted (Table 3). Consideration of the modeled scenario of the Essential LDHF Program activities could provide ways in which to affordably integrate and sustain LDHF training which may have more health impact per dollar spent than other MNH interventions, including alternative trainings.

### Comparison to other training alternatives

The most common alternative training is an offsite BEmONC classroom-based workshop; we conducted a cost analysis of such workshop trainings led by the GCNM in Accra and in Kumasi. The GCNM workshops targeted midwifery tutors from pre-service educational institutions across Ghana whereas Jhpiego’s LDHF program targeted midwives practicing in hospitals. For this reason, as well as other confounding variables relative to the assessments and aspects of the respective institutions, health effects of the traditional BEmONC training were not evaluated or pursued for inclusion in the cost-effectiveness analysis. Comparing program costs alone still contributes by clarifying differences in key program activities and the associated costs as well as measuring efficiency when considering total cost per trainee between the workshop and LDHF training. One of the major distinctions between LDHF and workshop-based approaches is that many staff present at the facility can be trained in LDHF whereas one or two representatives from a facility are typically trained in a workshop-based training.

Other new training methods for maternal, newborn, and child health care improvement have been studied, though few connect this training with associated health outcomes. One such program implemented in seven states of South Sudan had several critical components in common with Jhpiego’s LDHF training in Ghana: an 8-day training course for local trainers, a shorter course for trainees, use of mannequins for training, and reusable medical equipment left for newborn resuscitation and care. Over 2 years, this program trained 708 frontline health workers with a sustained improvement of 40.4% in maternal objective structured clinical examination (OSCE) scores and 3.1% in newborn OSCE scores at the time of 2-month follow-up and resulting increased referral rates [47]. Though not directly comparable to Jhpiego LDHF given differences in OSCE assessments, setting, and health worker cadre trained, this is a similar example of MNH training that saw positive results. Other studies have explicitly analyzed the viability of low-dose training: a Kenyan program held 1-day newborn resuscitation trainings for public hospital health workers and found that short onsite trainings had an immediate impact on practice and associated health measures [48].

### Limitations

The major limitation was a lack of a comparison group of facilities trained with the alternate approach, a BEmONC traditional workshop, in the same time period, for which the same health outcomes could be measured. Although a GCNM BEmONC workshop covered the same training content as the LDHF program during the same time period, the fundamental differences in facility and trainee characteristics rendered any comparison of facility health outcomes invalid. Jhpiego and the Ghana Health Service coordinated to minimize any MNH trainings that could be potential confounding factors to the impact of the LDHF approach; although a few sporadic trainings may have taken place, no trainings with hands-on practice, follow up, and mentoring were recorded during the timeframe [26]. Future research of LDHF in comparison to the traditional workshop-based approaches needs to go beyond cost comparison and consider health effects resulting from this distinct training alternative.

## Conclusion

The LDHF approach to training presents a unique opportunity to build lasting capacity for cadres of frontline health workers that currently receive only basic training. These are the same health providers who may not usually be selected for workshop training programs due to lack of experience or seniority in the facility; arguably, these frontline health workers have the most need to engage with trainers and are most likely to provide care to patients regularly. LDHF responds to this need and—quite literally—meets providers where they are in order and helps them achieve outstanding gains in knowledge and competency, retain that capacity, and use their skills and expertise in effectively improving MNH outcomes.

At $53.07 per DALY averted, this intervention is highly cost-effective when considered with several critical cost-effectiveness thresholds and represents good value for money in Ghana. To accelerate progress toward SDG 3, LDHF training for basic emergency newborn and obstetric care should be considered for adoption and scale-up by the GHS and other implementing partners. Additional modifications building on lessons learned from cost modeling have potential to make the LDHF training program more efficient in future implementations.

## Abbreviations

BEmONC: Basic Emergency Obstetric and Newborn Care
CI: Confidence Interval
DALY: Disability-adjusted Life Year
GCNM: Ghana College of Nurses and Midwives
GDP: Gross Domestic Product
GHS: Ghana Health Service
GNI: Gross National Income
LDHF: Low-Dose, High-Frequency
MM: Master Mentor
mMentoring: Mobile Mentoring
MNH: Maternal and Newborn Health
NGO: Nongovernmental Organization
OSCE: Objective Structured Clinical Examination
SDG: Sustainable Development Goal
